# A Simplified and Effective Method for Generation of Experimental Murine Periodontitis Model

**DOI:** 10.3389/fbioe.2020.00444

**Published:** 2020-05-25

**Authors:** Danfeng Li, Yi Feng, Hang Tang, Lijia Huang, Zhongchun Tong, Cheng Hu, Xiaodan Chen, Jiali Tan

**Affiliations:** ^1^Department of Orthodontics, Hospital of Stomatology, Guanghua School of Stomatology, Sun Yat-sen University, Guangzhou, China; ^2^Guangdong Provincial Key Laboratory of Stomatology, Sun Yat-sen University, Guangzhou, China

**Keywords:** periodontitis, bone loss, animal model, periodontal tissue defects, tissue engineering model

## Abstract

Periodontitis, a common disease that can lead to bone destruction, periodontal attachment loss, and tooth loss, is the major cause for oral tissue engineering. Experimental periodontitis is a suitable disease-model for studying bone regeneration and the potential therapeutic role of biomaterials on periodontal tissue engineering, as this *in vivo* model could be employed to mimic the natural host response under bacteria-caused oral pathological environments. Although large animals with ligature-induced periodontitis have mostly been used for experiments, a mouse model is a better choice for several reasons. Inserting ligature threads through the interproximal space between the teeth is the key step in establishing a periodontitis model, and it is easy to achieve in large animals, but difficult in mice due to the limited operating space. In this work, we provide a new and proven approach for periodontitis induction in mice using C+ nickel-titanium root canal files and stainless-steel ligature wires. The validity of this method was assessed by evaluating alveolar bone loss via micro-CT and detecting periodontal inflammation by histological staining and qPCR after the treatments. Progressive alveolar bone loss was observed from day 3 after the ligature-placement. Infiltration and accumulation of F4/80+ macrophage was also detected. In accordance with the histological results, there was upregulation of the expression levels of the inflammatory genes *Il1*β, *Tnf-*α, and *Il6* in gingival tissues isolated from the ligation sites. Our results suggest that this novel method could resolve the difficulty of ligature-placement in mice and consequently contribute to further use of mouse models for studying the pathological mechanisms of periodontitis and developing potential periodontal tissue regeneration strategies. C+ files, which are made of nickel-titanium, are tough, elastic, and sufficiently thin to pass through the interproximal space between the teeth after pre-bending to form an appropriate angle, thus providing an access for ligature wire insertion. As a common tool in the dental clinic, it is familiar to researchers of oral biology, and can provide the feasibility for wide application of our method.

## Introduction

Periodontitis is a chronic inflammatory disease caused by bacterial pathogens and is characterized by inflammatory infiltration and progressive alveolar bone loss (Hajishengallis et al., 2012), and it is highly prevalent all over the world (Lamont et al., 2018). In addition to being the major cause of bone destruction, periodontal attachment loss, and tooth loss in adults, periodontitis is associated with an increased risk of developing fatal systemic disorders such as atherosclerosis (Hajishengallis, 2015), hypertension (Schiffrin and Engert, 2019), rheumatoid arthritis (Potempa et al., 2017), and diabetes (Blasco-Baque et al., 2017; Xiao et al., 2017).

Previous studies on periodontitis therapy examined the clearance of pathogenic bacteria and usage of biomaterials for repairing periodontal defects (Ouchi and Nakagawa, 2020). Thus, there is a strong need to determine the most optimal preventive therapies for periodontitis and perform research on regenerative treatments using appropriate animal models (Liu et al., 2018; Kourtzelis et al., 2019). For example, Datey et al. used a rat periodontitis model and tested the effect of shockwave treatment in combination with antimicrobials on periodontitis (Datey et al., 2019). Apart from the significance of studying periodontitis itself, it is also a suitable model for studying bone regeneration and investigating the potential therapeutic functions (such as anti-inflammation, anti-osteoclast, and osteogenesis) of biomaterials on periodontal tissue engineering, as it is easily obtained and accessible for observation (Eskan et al., 2012; Manilay and Zouali, 2014; Tsukasaki et al., 2018).

Previous studies showed that the animal models of periodontal bone defects, formed by flap surgery and removing alveolar bone tissue with a dental bur, are one of the most widely used bone defect models in the study of periodontal bone reconstruction and regeneration (Nasajpour et al., 2018; Hasani-Sadrabadi et al., 2019). We have been engaged in the research on intervention strategies of periodontal tissue repair under periodontitis. We established a supramolecular hydrogel SDF-1/BMP2/NapFFY, which could effectively repair alveolar bone defects in rats. The above results were published in Tan et al. (2019). We anticipate that the biomaterials developed could replace bone transplantation in a clinical setting for repair of periodontal bone defects under periodontitis. Periodontitis is one of the most common diseases that can lead to alveolar bone defects in oral clinics, which is caused by periodontitis pathogens. Different from the bone defects caused by mechanical factors, periodontitis creates a more complex periodontal ecological environment. Therefore, compared with the animal models of alveolar bone defects constructed by mechanical methods, the animal models of alveolar bone defects under experimental periodontitis are more appropriate to be used to simulate the periodontal micro-environment of periodontitis.

The ligature model is one of the most commonly used models to induce periodontitis because the ligature around the molar accelerates the formation of bacterial-plaque and leads to progressive periodontal attachment loss (Marchesan et al., 2018). In the past decade, dog, rabbit, and rat animal models have been used to study the pathogenesis and mechanisms of periodontitis because oral surgery and ligation are easily accomplished (Bhattarai et al., 2016; Courbon et al., 2019). There is experimental demand for gene-editing strains and experiments with high-quality immunochemical detection reagents, and therefore, mouse models have been increasingly applied to study periodontitis (Offenbacher et al., 2018). Additionally, small animals cost less than larger animals, and it is convenient to maintain mice in specific pathogen-free (SPF) environments. It is the technical problem in inserting the ligature into the interproximal spaces between the molars of the mice that makes use of the mouse model unpopular, because the oral cavity and teeth of the mice are too narrow, and it is difficult for operators to clearly see and place ligatures. Researchers have attempted to implement various methods for ligature-induced periodontitis in mice (Marchesan et al., 2018). Even so, more simple and economical methods are expected to solve the operative difficulties in periodontitis induction.

Here, we carried out an effective method for experimental periodontitis induction using pre-bent C+ Ni-Ti canal files to form an angle that is adapted to the small size of the oral cavity and placing a 0.2-mm orthodontic ligature between the maxillary molars. In this work, we introduce the procedures for ligature placement in detail and present the data that verify the effectiveness of experimental periodontitis induction. This model is suitable for periodontitis induction and is expected to aid in the study of pathological mechanisms and potential therapeutic treatments in periodontitis. In addition to the mechanism of periodontitis, this model can be used for study on the properties of biomaterials and periodontal tissue engineering research.

## Materials and Methods

### Animals

Wild-type female C57BL/6 mice (6–8 weeks old, 18–22 g in weight) were purchased from the animal center of Sun Yat-sen University (Guangzhou, China). All animal-related treatments described in these experiments were approved by the Institutional Animal Care and Use Committee (IACUC) of Sun Yat-sen University. Mice were randomly divided into each group (sham or ligature insertion) and maintained in an SPF environment with a temperature of 22 ± 2°C, 55% humidity, light/dark cycle of 12/12 h, and standard food and water (provided by the animal center) *ad libitum*. Mice were euthanized at 0, 1, 3, 6, and 10 days after surgery followed by micro-computed tomography (CT), histological staining, and quantitative PCR (qPCR) studies. The subject was excluded if the inserted ligatures fell out 3 days after ligature-placement. The investigators were blinded to the group while the data measurements and outcome evaluations were being performed.

### Establishment of Experimental Periodontitis in a Murine Model

The previous method of ligature placement around the maxillary second molar is difficult to achieve in mice due to the limited operating space (Marchesan et al., 2018). We explored a simplified method to establish experimental periodontitis model by C+ Ni-Ti root canal files and 0.2-mm orthodontic ligature wires. Mice were anesthetized with an intraperitoneal injection of 1% pentobarbitone (dissolved in PBS, 100 μl/20 g) before the surgery to induce periodontitis. Experimental periodontitis was induced by C+ files (8#, 10#, or 15#, DENTSPLY Maillefer, Switzerland) and insertion of ligature wire (Orthodontic Stainless Ligature Wire, 0.2 mm in diameter) between the left maxillary molars, as described in detail in Table 1. The sham control mice received the same surgery but without wire-placement. The mice were monitored and kept warm until they recovered from the anesthesia. Every 3 days, the mice were anesthetized, and the presence of the ligature wire was evaluated. Animals in which the ligature wire was absent were excluded from the study. After the periodontitis induction, the mice were euthanized by an overdose of anesthesia.

**Table 1:** Detailed procedure of the modified method to induce experimental periodontitis.

**Step**	**Treatments (using the left side of the maxillary molars as an example)**	
1	Exposure of the operating view in the oral cavity	• Hold the curved tweezers with the left hand and point its curved tip upward to prop open the mandible of the mouse and prevent the tongue from falling down to obstruct the view
		• Tie an orthodontic ligature (6–8 cm in length) around the maxillary incisor and tape it to the operating table
2	Pretreatment with C+ files	• Pre-bend 5 mm of the tip of the 8#, 10#, or 15# C+ nickel-titanium root canal files
		• Hold the C+ file with the right hand and carefully use it as a probe to determine the interproximal region between the 1st and 2nd molar and the 2nd and 3rd molar
		• Slowly file into the adjacent space and gently pull back ~2 mm to form a path between the interproximal space. Then separate the filled gingival tissues, for the purpose of the following orthodontic ligature placement
		✧ The reason for the above operation: 1. Because of the flexibility of the orthodontic ligature, it is easily bent and deformed. Thus, it is difficult to insert into the interproximal space because of the blockage of gingival tissues. 2. Being a type of dental root canal file with both stiffness and elasticity, C+ nickel-titanium files are clinically used to locate the narrow orifice of calcified root canals. Because they are sufficiently thin and can pass through the interproximal space, and operators can determine correct placement by touch and feeling feedback, C+ root canal files enable access so that the orthodontic ligature can be successfully placed
3	Insertion of the orthodontic ligature wires	• Prepare the orthodontic ligature wires (0.20 mm in width, 3–5 mm in length). ✧ If the wire is too long, it could easily pierce the oral mucosa and enter the buccinator, which will adversely affect the ability of mice to eat and could inhibit adequate food intake. Furthermore, a wire that is too long will be easily licked and removed by the tongue, causing it to fall off from the interproximal space. Conversely, if the ligature is too short, it is difficult to clamp and bend it into an appropriate angle for further insertion
		• Slightly bend the orthodontic ligature wire into a “(” shape, in order to form a proper angle for subsequent insertion
		• Hold the needle holder with the right hand, and clamp the ligature with the tip of the needle holder
		• Gently probe the adjacent region prepared by the root canal files above with the orthodontic ligature to determine the correct location, and then gently insert the orthodontic ligature into the interproximal space
4	Completion	• Slightly move the ligature with tweezers to repeatedly check whether the ligature has been properly placed

### Micro-CT Analysis

Maxillary samples were collected at 0, 1, 3, 6, and 10 days (Jiao et al., 2013) after surgery and scanned with a Scanco μCT50 scanner (Scanco Medical AG, Brutishly, Switzerland) with a resolution of 20 μm at 70 kVp and 200 μA. The 3D images from the buccal and palatal sides were constructed using Materialize Mimics v17.0 software. As for bone loss evaluation, the distance between the cemento-enamel junction (CEJ) and alveolar bone crest (ABC) was measured by the abovementioned software. Twelve sites from each maxillary molar were chosen for measurement, and averaged distances were calculated. Each measurement was repeated three times.

### Histological Analysis

The maxillae were dissected at 0, 3, 6, and 10 days after experimental periodontitis treatment and were subsequently fixed with 4% paraformaldehyde. For tissue immunofluorescent staining, the maxillae were then decalcified with 0.5 M ethylenediaminetetraacetic acid (EDTA) for 48 h and embedded at −80°C with compound (OCT, Sakura Finetek, Torrance, CA, USA). Frozen sections were sliced at −25°C and stored at −20°C. After antigen repairing and blocking for 1 h at room temperature in 3% bovine serum albumin (BSA), the slices were incubated overnight using the primary antibody of rat-anti-mouse anti-F4/80 (Abcam Cat# ab16911, RRID:AB_443548). Then, goat-anti-rat Alexa Fluor® 488 secondary antibodies (Abcam Cat#ab150157, RRID:AB_2722511) were used at 1/200 dilution to further stain the slices on the following day. The slices were counterstained with Hoechst 33342 Staining Dye Solution (ab228551) for 10 min at 25°C. Laser-scanning confocal microscopy was employed to detect the fluorescent distribution.

### RNA Extraction and qPCR Analysis

At 0, 3, 6, and 10 days after the wire insertion, total RNA was isolated and purified from the gingival tissues surrounding three maxillary molars via a series of centrifugation and extraction treatments using TRIzol reagent, chloroform, isopropanol, and 75% diethyl pyrocarbonate (DEPC)-ethanol. First-strand cDNA was synthesized with 1 μg of total RNA and an oligo (dT) primer using a commercial kit (All-in-One cDNA Synthesis SuperMix, Cat: B24403, Bimake) according to the manufacturer's instructions. The expression levels of inflammatory genes including *Il1b, Tnf-*α, and *Il6* were detected by qPCR using the Roche Real-Time PCR System with SYBR Green (FastStart Essential DNA Green Master, Cat: 35732800, Roche, USA). The detection was independently replicated twice. The expression levels were normalized by β-actin mRNA level.

### Statistical Analyses

Differences between multiple groups were evaluated using one-way-ANOVA analysis (Tukey's multiple comparisons test). Differences at *P* < 0.05 were considered significant. Statistical analyses were performed using the GraphPad Prism software version 7.0 (GraphPad Software, La Jolla, CA, USA).

## Results

### Morphometric Evaluation of Alveolar Bone Loss After Experimental Periodontitis Induction

**Figure 1:**
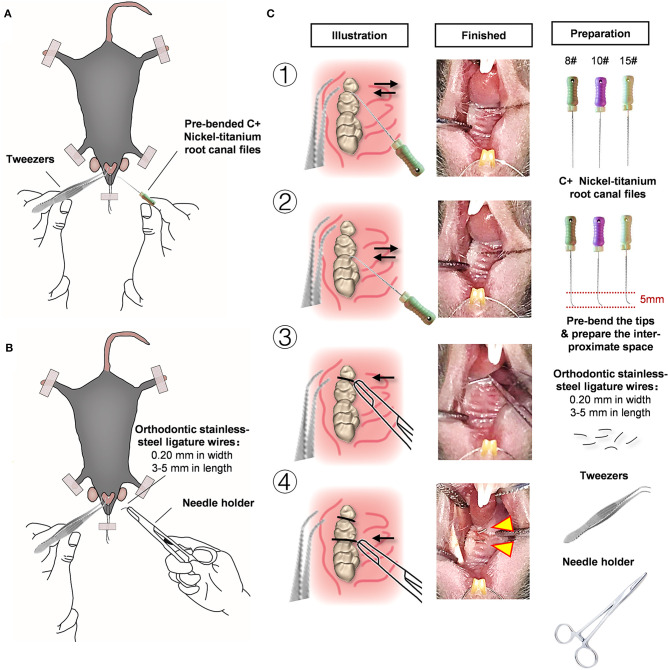
An effective and simple experimental mice model for periodontitis research. **(A)** Pre-bend the 5 mm tip area of the C+ nickel-titanium root canal file, then slowly file into the adjacent space. **(B)** Orthodontic ligature wires (cut by 0.2 mm in width, 3–5 mm in length) were inserted through interdentium between the first molar and second molar, and the second molar and third molar using a needle holder. **(C)** Detailed procedures and preparation of the modified wire-placement treatment.

For experimental periodontitis induction, the tip of a C+ nickel-titanium root canal file was pre-bent and slowly filed into the interproximal space between the maxillary molars (Figure 1A). The orthodontic ligatures (0.20 mm in width, 3–5 mm in length) were inserted (Figure 1B), as shown in the detailed procedure illustrated in Figure 1C. Furthermore, we present a simple method for fixing the mice to the operating table and exposing the oral cavity using orthodontic ligature wire. More importantly, the average operative time for the model by five independent operators is shown in Supplementary Figure 1. After practicing three times with mice exhibiting different health conditions, the operators were able to successfully place ligature wires on one side of the maxillary molars in less than 10 min, which saved a lot of time. This method is simple and easy to conduct.

**Figure 2:**
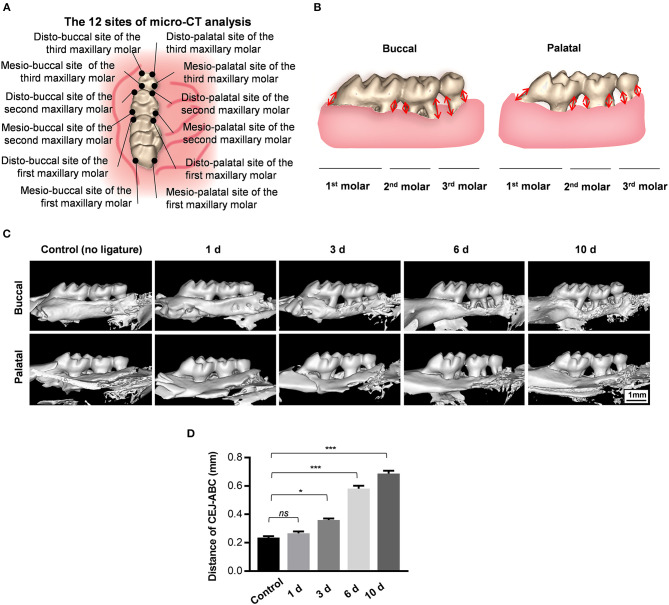
Orthodontic ligature wire insertion induces progressive alveolar bone loss. **(A)** Anatomical direction of maxillary molars. **(B)** Schematics of strategy for bone loss measurement by assessing the distances from the cementoenamel junctions to the alveolar bone crest (CEJ-ABC) on buccal and palatal side. **(C)** Representative sagittal 3D images viewed from the buccal side and palatal side of the maxillary molars 0, 1, 3, 6, and 10 d after ligature-insertion. **(D)** Statistical analysis of the linear distance of CEJ-ABC 0, 1, 3, 6, and 10 d after experimental periodontitis operation. Data are shown as mean ± SD. (*P < 0.05, ***P < 0.001, ns, not significant, *n* = 3, One-way ANOVA, Tukey's multiple comparisons test).

To analyze alveolar bone loss, the distances between the cemento-enamel junction (CEJ) and alveolar bone crest (ABC) were measured at four sites for each maxillary molar (both palatal and buccal, distal and mesial) in three-dimensionally constructed images. The mean distances for 12 sites from each sample were calculated and are presented in millimeters (Figures 2A,B). Both the buccal side and the palatal side were used for evaluation of ligature-induced bone loss in order to reliably assess the damage to the alveolar bone. Morphological alterations in buccal and palatal alveolar bone caused by the progression of wire-induced periodontitis are visually presented in 3D-constructed images, showing prominent bone loss at the mesial and distal sites of the second molar 3 days after periodontitis induction (Figure 2C). As shown, the specimens from the experimental periodontitis groups displayed abrupt aggravation of bone loss from day 3 to day 6 and relatively smooth progression from day 6 to day 10 by assessing the CEJ-ABC distances of 12 sites in the palatal and buccal surfaces.

Notably, the CEJ-ABC distance values in the 1d-group were similar to those of the control group that received no ligatures, indicating that the observed progressive alveolar bone loss could hardly be caused by operative damage caused by placement of the orthodontic wire itself (Figure 2D). Furthermore, the average CEJ-ABC distance in the control group was 0.24 ± 0.01 mm, which is larger than the thickness of the orthodontic ligature wire (0.2 mm), indicating that the alveolar bone loss might not be caused by the trauma of wire-insertion. In sum, these data demonstrate that this new method of orthodontic wire placement can simulate progressive bone loss and is appropriate for establishing experimental periodontitis.

### Inflammatory Infiltration in Gingival Tissue After Ligature Insertion

A predominance of macrophage infiltration has been observed in the lower region of chronic periodontitis lesions (Kourtzelis et al., 2019; Zhuang et al., 2019). Among the immune cells, macrophages are intensively studied because they might respond to microbial pathogens and further activate the adaptive immune response.

**Figure 3:**
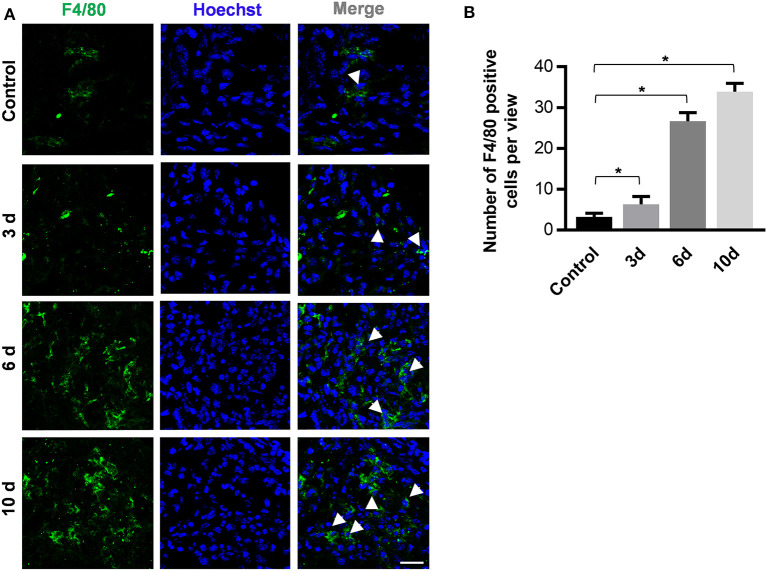
The periodontitis induction method of orthodontic wire insertion aggravates macrophage infiltration in periodontal tissues. **(A)** Sliced sections of periodontal tissues which were collected at 0, 3, 6, and 10 days after wire-placement. The interproximal space between the first molar and the second molar is observed. Representative confocal images of stained periodontal tissues. Arrowheads mark F4/80+ cells. (scale bar, 25 μm). **(B)**The number of F4/80 positive cells in the view of **(A)** between the first and the second molars were calculated at all time points. Data are shown as mean ± SD. *P < 0.05. (*n* = 3, One-way ANOVA, Tukey's multiple comparisons test).

Slices of sagittal molar regions were prepared, and the areas between the first and the second molar were observed by confocal microscopy to detect infiltrated immune cells (Figure 3A). As the data show, there were increasing numbers of F4/80+ macrophages in the periodontal tissue between the first molar and the second molar 6 days after periodontitis induction (Figure 3B). These results indicate that immune cell infiltration occur and also illustrate the potential role of macrophages in the development of periodontitis.

### Differential Expression of Inflammatory Genes Throughout the Duration of Periodontitis

**Figure 4:**
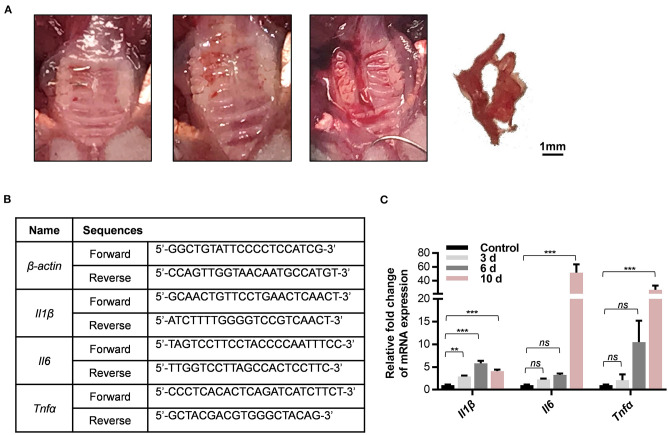
The new method for periodontitis induction increases expression of inflammatory gene in periodontal tissues. **(A)** Collection of gingival tissues around the ligature side of maxillary molars. **(B)** Sequences of gene-specific primers used in qPCR analysis. **(C)** The mRNA levels of *Il1*β, *Il6*, and *Tnf*α in whole gingival tissues including on 0, 3, 6, and 10 days after ligature-placed sites were determined by qPCR experiments. Results are shown as fold increase relative to the control tissues without experimental periodontitis treatment in mean ± SD. (**P < 0.01, ***P < 0.001, ns, not significant, *n* = 3, One-way ANOVA, Tukey's multiple comparisons test).

The gingival tissues in the molar region were collected after periodontitis treatments (Figure 4A). The mRNA levels of the inflammatory genes *Tnf*α, *Il1*β, and *Il6* were detected on different days after ligature-insertion [baseline (0), 3, 6, and 10 d]. As expected, all three inflammatory genes were upregulated after wire insertion as compared to the control group. The expression level of *Il1*β significantly increased 3 days after the periodontitis operation, which reflects the two well-known cytokines that are characteristic of inflammation in periodontitis and are universally used as detection markers in the acute phase of chronic inflammation.

In accordance with the histological data, the expression of the inflammatory genes *Il6* and *Tnf*α markedly increased 10 days after ligature insertion compared with baseline (Figure 4B). Apart from being a protective response to the accumulated microbial pathogens, this increase could trigger the downstream molecular signals that are recognized to accelerate inflammatory disease progression. The primers that were used in the qPCR experiments are listed in Figure 4C. Overall, the results suggest that this model could be appropriate for host response research during periodontitis duration. These results indicated that a simplified orthodontic ligature-induced periodontitis model in mice was successfully established to mimic the inflammation that occurs in the periodontium.

## Discussion

Periodontitis is a highly prevalent disease that leads to periodontal attachment loss, bone destruction, tooth loss, and systemic diseases. Experimental periodontitis is a suitable model for exploring anti-inflammatory, osteogenic, and anti-osteoclast characteristics of biomaterials in bone regeneration, as it is easy to create and is accessible for observation. The application of animal models has been limited by several factors, such as small oral cavity size, operative difficulty, and expense (Suzuki et al., 2006). Mice are the most ideal animal models for pathological and therapeutic research of periodontitis, because the mouse-derived antigen of the experimental specimen can be detected sensitively with high-quality immunochemical detection reagents. Furthermore, the cost of experiments with small animals is lower than that of large animals. In addition, compared with large animals, it is more convenient to keep mice in SPF environments for long-period researches.

According to the traditional method, inserting the ligature threads through the interproximal space between the 2nd and 3rd molars and the interproximal space between the 1st and 2nd molars is the key step to establishing the periodontitis model, and it is easy to achieve in large animals, but difficult in mice due to the limited operating space. As murine molars have a size range of 0.4–1.2 mm^2^, the traditional ligature placement around the maxillary second molar requires proficient technical skill (Marchesan et al., 2018). Furthermore, it is difficult to insert 0.2-mm stainless steel orthodontic ligature wire into the interproximal space because of the blockage of gingival tissues, which creates a barrier. Although 0.2-mm stainless steel orthodontic ligature wire is sufficiently durable to pass through the interproximal space and could promote bacterial accumulation (Alves de Souza et al., 2008), orthodontic wires are easily bent and deformed. In this work, we provide a unique perspective on a new method using orthodontic ligature wires and C+ Nickel titanium root canal files.

C+ nickel-titanium root canal files are stiff and durable, yet elastic. They have been used by dental operators to probe the narrow orifice of calcified root canals and determine their location via tactile feedback, which will indicate the sites of the tiny interproximal space between molars. An appropriate angle can be formed in pre-bent C+ nickel titanium root canal files, which are sufficiently thin and can pass through the interproximal space to assist with identifying the blockage of gingival tissues, thus providing an access point for the insertion of orthodontic ligature wires. To the best of our knowledge, we are the first to report a method for initiating experimental periodontitis using C+ nickel-titanium root canal files and orthodontic ligature wires.

Accordingly, micro-CT, histological staining, and inflammatory gene detection by qPCR were used to confirm the effect of experimental periodontitis induction. As the results show, the new approach simulates progressive bone loss and accelerates inflammatory infiltration. Hajishengallis et al. reported that the placement of ligature threads in mice caused alveolar bone loss by the natural accumulation of bacteria but not by mechanical trauma (Abe and Hajishengallis, 2013). Studies also reported that endogenous microorganisms could lead to periodontitis in ligation-treated mice (Abe and Hajishengallis, 2013; Jiao et al., 2013). Similar to the ligature threads that are used in the ligature model, orthodontic ligature wires, which are applied in dental treatments, provide an ideal habitat for the oral bacteria that cause periodontal diseases (Chun et al., 2007). As reported, orthodontic wire-induced oral plaque is the major cause for periodontal inflammation in orthodontic treatments (Liu et al., 2011).

Our data also verified that insertion of 0.20-mm orthodontic ligatures would not harm the alveolar bone or gingival tissue because no bone loss was observed in micro-CT analysis 1 day after wire placement. The results revealed that sustaining the successful periodontitis phenotype was dependent on accumulation of an endogenous oral microbiota rather than as a result of operative trauma to the periodontium. Like previously reported ligature models (Liu et al., 2018; Kourtzelis et al., 2019), our orthodontic-wire-induced gingival inflammation and alveolar bone loss but did not result in robust inflammation or bone damage. We took the study by Jiao et al. (2013) for reference, which applied the mice model by 10-day ligature placement. And the studies have induced alveolar bone loss and inflammatory infiltration, which represent the successful establishment of periodontitis. We decided to execute the mice 10 days after periodontitis induction so as to prove that our method could promote alveolar bone loss as effectively as the traditional one. Thus, this method can be used to mimic alveolar bone loss and gingival inflammation in periodontitis with minimal assistance and is appropriate for establishing experimental periodontitis.

The most noteworthy advantage of our method is that the tools of our presented method are economic and feasible. Focusing on the same problem, Marchesan et al. recently developed an approach to place ligature threads between molars, using an appliance they designed by 3D-printing technique (Marchesan et al., 2018). In this work, we provide a new and proven method using orthodontic ligature wires and C+ Nickel titanium root canal files. These two tools are common in dental clinics but not usual in other institutes. The other researchers may probably not be as familiar with these tools as the dental researchers. However, since using the C+ Ni-Ti root canal files and orthodontic ligature wires is economic and feasible, more researchers could learn this simple method and apply it to their future research after reading this work. More importantly, our facile method can be conducted by a single operator without assistance and in less than 10 min after practicing, thus saving much time. Our modified wire-induced periodontitis model can be used in conjunction with periodontal pathogens to mimic the host response and develop anti-bacterial therapy. The short operative time of our method is advantageous for the survival of exogenous anaerobic periodontal pathogens.

We think this technique can be further used in rats, rabbits, dogs, goats, or larger animals by using thicker ligature wires and larger sizes of C+ Ni-Ti root canal files. The creative technique can greatly improve research efficiency, success rate, and technical limitations. In addition to pathological mechanism research, experimental periodontitis is a suitable disease model for studying bone regeneration and elucidating the potential therapeutic role of biomaterials on periodontal tissue engineering. Once serious alveolar bone loss has occurred, it is difficult to achieve restoration and regeneration. Tao et al. developed an injectable woven bone-like hydrogel to help alveolar ridge bone remodeling after tooth extraction, so as to preserve enough bone for the insertion of dental implants (Yang et al., 2020). In oral clinic, it is common that periodontitis leads to alveolar bone loss, tooth loosening, and falling off, which require dental implant treatment. Establishing the animal model mimicking the bone microenvironment is becoming increasingly necessary to explore bone substitute biomaterials. This *in vivo* periodontitis model could be used preclinically to investigate potential tissue engineering treatments under bacteria-caused pathological oral environments. Using this model to test the antibacterial, anti-inflammatory, and osteogenic properties of biomaterials under the condition of periodontitis will help to verify the therapeutic effect of biomaterials on alveolar bone defects caused by periodontitis, so as to provide evidence for their future clinical applications. What is more, our study inspired an enlightening thought that we can create novel and efficient methods for establishing traditional animal models through multidisciplinary collaboration. Cross-subject research is a new trend in the future. This technique is a new combination of clinical endodontics and basic periodontological research.

In conclusion, we explored a novel approach to generating a mouse model of periodontitis induction. The results showed that progressive alveolar bone loss and periodontal inflammation were observed after placement of orthodontic ligature wires. Therefore, our model could be suitable for studying the pathogenesis of periodontitis, the mechanism of periodontal defects, and regenerative treatments.

## Data Availability Statement

The datasets generated for this study are available on request to the corresponding author.

## Ethics Statement

The animal study was reviewed and approved by Institutional Animal Care and Use Committee (IACUC), Sun Yat-sen University (Approval No. SYSU-IACUC-2020-000146).

## Author Contributions

DL performed the research, analyzed data, and wrote the paper. YF participated in the research and wrote the paper. ZT, LH, and CH assisted in the animal experiments, sample preparation, and consultation of periodontitis bacteria. HT and XC involved in the routine management, procurement of laboratory and the study. JT conceived of and organized the research. DL and YF contributed equally in this work. All authors contributed to manuscript revision, read and approved the submitted version.

## Conflict of Interest

The authors declare that the research was conducted in the absence of any commercial or financial relationships that could be construed as a potential conflict of interest.
