# Supplementary figures and images for: A Simplified and Effective Method for Generation of Experimental Murine Periodontitis Model
Source: Front Bioeng Biotechnol. 2020 May 25;8:444. doi: 10.3389/fbioe.2020.00444 (PMC7261919; doi:10.3389/fbioe.2020.00444)

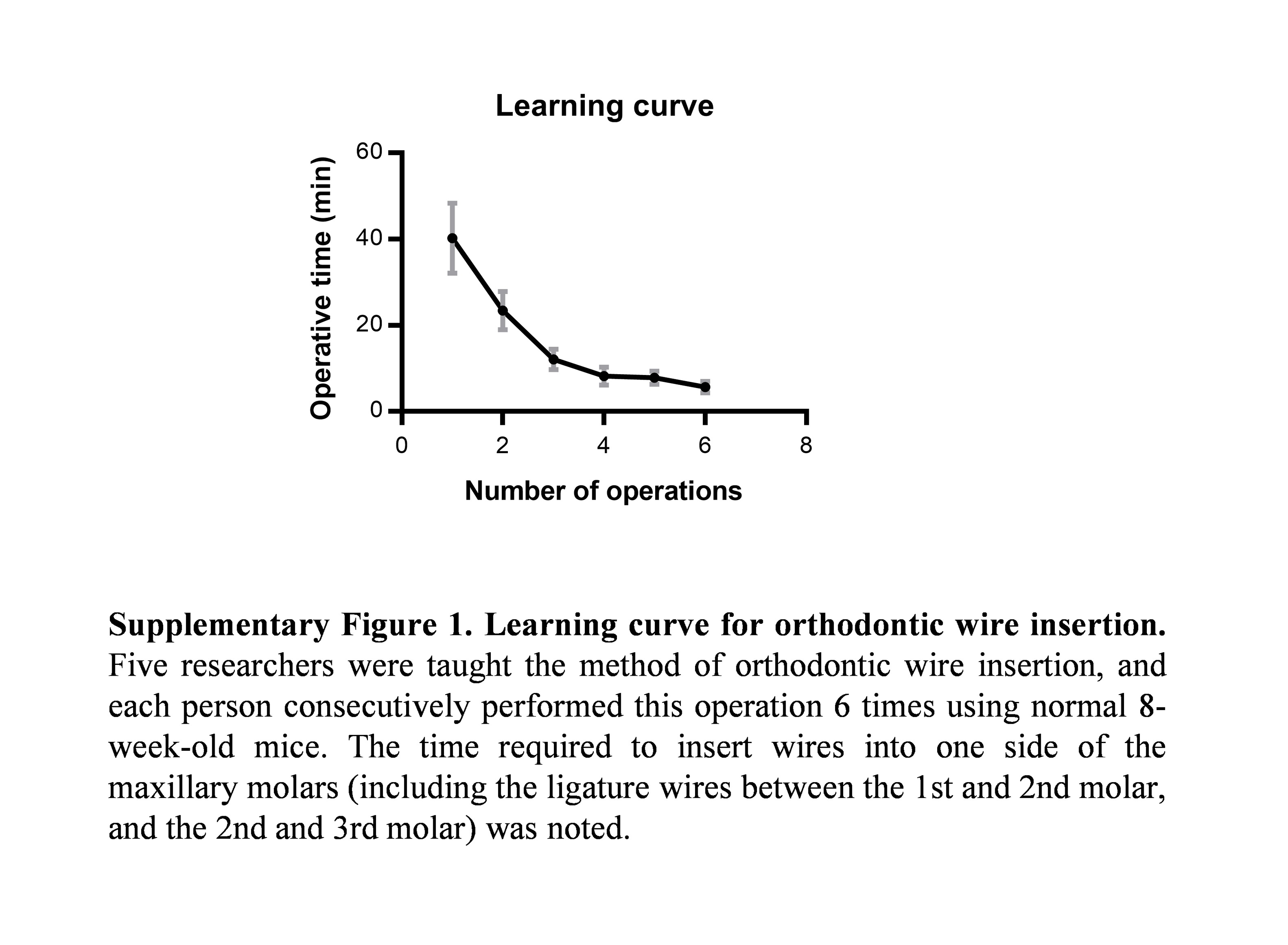

Supplement: Supplementary file 1 [file Image_1.TIF]
